# Music prevents stress-induced depression and anxiety-like behavior in mice

**DOI:** 10.1038/s41398-023-02606-z

**Published:** 2023-10-12

**Authors:** Qiang Fu, Rui Qiu, Lei Chen, Yuewen Chen, Wen Qi, Yong Cheng

**Affiliations:** 1 Institute of National Security, Center on Translational Neuroscience, Minzu University of China, Beijing, China; 2 School of Ethnology and Sociology, Minzu University of China, Beijing, China; 3 College of Life and Environmental Sciences, Minzu University of China, Beijing, China; 4 Chinese Academy of Sciences Key Laboratory of Brain Connectome and Manipulation, Shenzhen Key Laboratory of Translational Research for Brain Diseases, The Brain Cognition and Brain Disease Institute, Shenzhen Institute of Advanced Technology, Chinese Academy of Sciences; Shenzhen–Hong Kong Institute of Brain Science–Shenzhen Fundamental Research Institutions, Shenzhen, Guangdong 518055 China; 5 Guangdong Provincial Key Laboratory of Brain Science, Disease and Drug Development, HKUST Shenzhen Research Institute, Shenzhen, Guangdong 518057 China; 6 College of Dance, Minzu University of China, Beijing, China

**Keywords:** Molecular neuroscience, Scientific community, Depression

## Abstract

Depression is the most prevalent psychiatric disorder worldwide and remains incurable; however, there is little research on its prevention. The leading cause of depression is stress, and music has been hypothesized to alleviate stress. To examine the potential beneficial effects of music on stress and depression, we subjected mice to chronic unpredictable mild stress (CUMS) during the day and music at night. Strikingly, our results indicated that music completely prevented CUMS-induced depression and anxiety-like behaviors in mice, as assessed by the open field, tail suspension, sucrose preference, novelty suppressed feeding, and elevated plus maze tests. We found that listening to music restored serum corticosterone levels in CUMS mice, which may contribute to the beneficial effects of music on the mouse brain, including the restoration of BDNF and Bcl-2 levels. Furthermore, listening to music prevented CUMS-induced oxidative stress in the serum, prefrontal cortex, and hippocampus of mice. Moreover, the CUMS-induced inflammatory responses in the prefrontal cortex and hippocampus of mice were prevented by listening to music. Taken together, we have demonstrated for the first time in mice experiments that listening to music prevents stress-induced depression and anxiety-like behaviors in mice. Music may restore hypothalamus-pituitary-adrenal axis homeostasis, preventing oxidative stress, inflammation, and neurotrophic factor deficits, which had led to the observed phenotypes in CUMS mice.

## Introduction

The 21st century is an era of global economic development, strengthening of scientific power [1], and fierce competition. The working population is facing hardships, helplessness, competition, pressure, worries, and depression, and the number of people with depression is increasing [2]. Depression severely impairs psychosocial function and reduces the quality of life [3, 4]. Due to the burden of work and duties, many people experience chronic unpredictable stress. It is estimated that depression will account for the highest number of loss of healthy life years because of a disability by 2030 worldwide [5]. Up to 25% of the world’s population is affected by depression and pathological anxiety, which impose a substantial health burden on the contemporary society [6].

Depression has an unknown etiology and complex symptoms [7]. In recent years, the incidence of depression has been increasing annually with an increase in social pressure and an accelerated pace of life [8]. Its common clinical manifestations include obvious anxiety, anhedonia, pessimism, and suicidal tendency [9, 10]. Despite its complex mechanisms, increasing evidence has identified the involvement of neurotrophic factors, inflammatory cytokines, the hypothalamus-pituitary-adrenal (HPA) axis, and oxidative stress/anti-oxidative stress systems in the pathophysiology [11–13]. HPA-axis disorders play a key role in the development of depression. Clinical tests have shown that depression is often accompanied by hyperactivation of the HPA axis, which is mainly characterized by increased cortisol levels [14–17].

Although current treatments for depression include chemicals, peptides, and gene therapies, nearly one-third of patients with depression are insensitive to existing drugs [18–21]. Moreover, the blood-brain barrier limits most drugs from reaching the brain, leading to unsatisfactory therapeutic outcomes [22]. Current regimens used to treat depression have many adverse effects and a negative impact on patient health. Therefore, there is an urgent need to develop alternative therapies with a higher efficacy and fewer side effects [23].

The 21st century is an era of high admiration for pure art [24]. In the context of globalization, art is the fusion, projection, and reflection of cultures, contexts, things, self, history, and civilization process. Since the beginning of the 21st century, owing to the rapid development of technology, increased productivity, economic integration, and mutual influence of exchanges between different cultural backgrounds, art, in the process of globalization, has increasingly assumed a contemporary character that resonates greatly with the present generation. Music is an art that reflects the real-life emotions of human beings. It is an artistic language that expresses or holds people’s feelings, conveys emotions more directly than ordinary language, and is associated with beauty [25]. Music therapy treats patients with physiological or psychosocial diseases using rhythms and tones, and is one of the most effective methods of spiritual healing for psychosomatic disorders [26]. Music can help patients better express their emotions and promote the communication of inner emotions [26, 27]. It can be a good preventative measure against prenatal depression [28, 29]. Music therapy is often used as an adjunct in the treatment of depression and has become widely popular in developed countries such as the United States [30, 31]. However, limited research has been conducted on the mechanisms by which music prevents depression. We analyzed the effect of music on a mouse model of chronic unpredictable mild stress (CUMS) using biomedical methods to explore its possible mechanism of action in preventing depression.

## Materials and methods

### Animal preparation

Male C57BL/6 mice (7 weeks old, 22 ± 1.5 g) were purchased from Fang Yuanyuan Breeding Farm (Beijing, China). The animals were raised at 23 ± 1 °C and 50 ± 1% relative humidity under a 12-h light/dark cycle (lights on from 8 a.m. to 8 p.m.) and provided ad libitum access to a standard diet and drinking water. All animal experiments in this study were conducted in accordance with the National Institutes of Health Laboratory Animal Care and Use Guidelines (NIH Publication No. 80-23) and approved by the Animal Care and Use Committee of Minzu University of China. We divided the mice into four groups, control (*n* = 6), CUMS (*n* = 10), Music (*n* = 6), and CUMS+Music (*n* = 14), respectively.

### Music and CUMS induction

CUMS was induced as previously described [32]. Briefly, CUMS mice were induced by the use of several stressors including restraint (4 h), cage tilt (45° for 24 h), wet bedding (24 h), food and/or water deprivation (24 h), tail nip (1 cm from the end of the tail for 3 min), cold water swimming (4 °C for 3 min), and light inversion (24 h). Mice were housed individually and exposed to three different stressors daily.

CUMS+Music mice listened to music for 1.5 h each night from 20:30 to 22:00. The music player was located approximately 2 m away from the mice, and music was played in a random order from the song list to simulate the listening pattern of the normal population. Regarding the selection of music, in the research endeavor undertaken by the team specializing in music studies, a carefully curated collection of 25 musical pieces (detailed music frequency see Table 1) was assembled to facilitate auditory stimulation experiments involving murine subjects. The compilation exhibits a comprehensive range of musical styles that extend across various historical periods, encompassing the Baroque, Classical, and Romantic eras. As for musical genres, the assortment presents a diverse selection of Eastern and Western instrumental and vocal compositions, integrating instruments such as the piano, flute, harp, violin, guqin, and Cucurbit flute. The vocal repertoire comprises operatic arias, indigenous folk songs, and contemporary popular tunes, with a particular emphasis placed on the rich legacy of traditional Chinese ethnic and folk music originating from a multitude of cultural groups, including Han, Tibetan, Mongolian, Dai, and Uighur.

**Table 1:** Music list.

No.	Composation	Composer	Genre	Time (s)	Frequency (Hz)
1	Piano Concerto No.23 in A Major, K.488	Wolfgang Amadeus Mozart	Piano	341	172–20,112
2	Melodie	Christopf Willibald Gluck	Violin	196	215–18,992
3	Serenade in G major, K525	Wolfgang Amadeus Mozart	String quartet	322	215–20,241
4	Tempo di Minuetto in the Style of Gaetano Pugnani	Fritz Kreisler	Violin	216	388–20,284
5	Romantic Pieces, Op.75 No.1	Antonín Leopold Dvořák	Violin	194	215–22,006
6	Greensleeves	King Henry VIII	Harp	245	258–11,585
7	12 Variations on “Ah, vous dirai-je Maman” in C major K.265	Wolfgang Amadeus Mozart	Piano	277	388–19,165
8	Hearing the soft rain amid the mountains	Yi Su	Guqin	332	234–20,531
9	The Nightingale	Alexander Aleksandrovich Alyabyev	Song	271	86–20,069
10	Summer	Joe Hisaishi	Piano	153	258–11,413
11	Down By the Salley Gardens	Joanie Madden	Flute	229	215–20,370
12	Improvisation for March of Time	Eddie Condon	Jazz	182	172–20,112
13	Skylines at dawn	Nameless	Piano	217	215–16,021
14	The eternity of the moment	Haiyang Zhao	Piano	161	43–7235
15	Silver stars and milk way in the mind	CMJ	Piano	220	94–20,578
16	Amid brooks in deep mountains	Yi Su	Piano	228	188–19,734
17	Songs of the moon	Nameless	Song	208	172–20,370
18	Mother in dream	Lag Basuron	Song	225	258–20,284
19	Moonlight shining on you	Nameless	Song	171	345–17,097
20	Fernleaf Hedge Bamboo in the moonlight	Guangnan Shi	Cucurbit flute	277	86–20,155
21	Melancholia or mirth	July	Piano	234	172–20,112
22	Ballade pour Adeline	Paul de Senneville	Piano	158	43–20,112
23	Nocturne in G Major, Op.37 No.2	Fryderyk Franciszek Chopin	Piano	398	172–5685
24	Waltz, Op. 70 No.1 in G-Flat Major	Fryderyk Franciszek Chopin	Piano	114	345–20,112
25	Prelude in C Major, BWV846	Johann Sebastian Bach	Piano	147	172–20,155

### Behavioral tests

The second day after the mice were treated with CUMS and/or music for 28 days, they were subjected to behavioral tests. Behavioral tests were conducted in a quiet environment and scored by the same researcher. The mice were transferred to the testing room at least 3 h before the behavioral tests.

#### Sucrose preference test

A sucrose preference test (SPT) was used to test the preference of mice for sugar to evaluate anhedonia [33]. Briefly, the mice were exposed to one bottle of 1% (w/v) sucrose solution for three days to habituate them to the solution. The mice were then exposed to both tap water and sucrose solution bottles for 24 h to obtain a sucrose preference baseline. Finally, the mice were subjected to a 12 h SPT, in which tap water and sucrose solution were provided in identical bottles. The positions of the two bottles were switched every 6 h and sucrose and water consumptions were measured simultaneously. The preference for sucrose solution consumption was calculated as percentage preference = [(sucrose intake/total intake) × 100]. The tests were performed by an individual who was blinded to the treatment status of the animals.

#### Open field test

An open field test (OFT) was used to assess the locomotor and exploratory behaviors of the mice [34]. The open field apparatus was divided into 16 equal squares. The mice were placed in the center. After a 2 min habituation period, the total movement distance, time spent in the center, and number of times the mice crossed the center were recorded for 3 min. The tests were performed by an individual who was blinded to the treatment status of the animals.

#### Tail suspension test

A tail suspension test (TST) was used to assess for the presence of despair/depression-like behaviors in mice [35]. The mice were suspended from the ceiling of a box with an adhesive tape placed approximately 1 cm below the tail tip. After a 1 min habituation period, immobility duration was measured for 5 min. If the time exceeds 5 min, it is also recorded as 5 min. The tests were performed by an individual who was blinded to the treatment status of the animals.

#### Novelty-suppressed feeding

The mice were starved for 24 h before being subjected to the test [36]. A white filter paper was placed in the middle of the testing device (50 × 50 × 45 cm), and food particles were placed on the paper. Individual mice were positioned on the corner grid of the floor of the testing device and allowed to roam at will. The period of inactivity before the mice started eating was recorded.

#### Elevated plus maze

The elevated plus maze (EPM) is one of the most commonly used rodent tests for assessing anxiety-like behavior, as described in our previous study [37]. The apparatus comprises two opposing open arms (50 × 10 cm) and two opposing wall-enclosed arms (50 × 10 × 40 cm). The maze is placed on a 50 cm high pedestal. The animals were acclimatized to the testing room for 30–45 min before the experiment. Each rat was placed on the central platform facing one of the open arms. The number of entries into the open and closed arms and the time spent in each arm in 5 min were recorded by an observer blinded to the animal groups. After each trial, the apparatus was wiped with 30% isopropanol to avoid odor effects.

### Quantitative real-time polymerase chain reaction

Total RNA was extracted from the hippocampus and prefrontal cortex of the mice using TRIzol reagent (Invitrogen, #15596026) [38]. Quantitative real-time polymerase chain reaction (qRT-PCR) was performed using 2×SYBR Green qPCR Master Mix (#Q341; Vazyme) in the StepOnePlus instrument (Applied Biosystems). The qPCR primer sequences used are listed in Table 2.

**Table 2:** Gene-specific primers for qRT-PCR.

Gene Name	Sequence (5′–3′)
IL-6-Forward	TGGCTAAGGACCAAGACCATCCAA
IL-6-Reverse	AACGCACTAGGTTTGCCGAGTAGA
IL-10-Forward	CCAAGGTGTCTACAAGGCCA
IL-10-Reverse	GCTCTGTCTAGGTCCTGGAGT
IL-1β-Forward	AGCTGGAGAGTGTGGATCCC
IL-1β-Reverse	CCTGTCTTGGCCGAGGACTA
TNF-α-Forward	GGCTTTCCGAATTCACTGGAG
TNF-α-Reverse	CCCCGGCCTTCCAAATAAA
TGF-β-Forward	GTGGAAGATTACAAGCCACCA
TGF-β-Reverse	GGGTCTGAGAACCATCTGTTAGG
iNOS-Forward	CAGCTGGGCTGTACAAACCTT
iNOS-Reverse	CATTGGAAGTGAAGCGTTTCG
Bcl2-Forward	GGCCTTCTTTGAGTTCGGTG
Bcl2-Reverse	GCATGCTGGGGCCATATAGTT
Bax-Forward	TGCTAGCAAACTGGTGCTCA
Bax-Reverse	CTTGGATCCAGACAAGCAGC
BDNF-Forward	GGTCTGACGACGACATCACT
BDNF-Reverse	TAGAGGAGGCTCCAAAGGCA
Actin-Forward	AGACCTCTATGCCAACACAGT
Actin-Reverse	TCCTGCTTGCTGATCCACAT

### Detection of oxidative markers

Oxidative markers were detected using the enzymatic colorimetric test following the manufacturer’s instructions. Briefly, peripheral serum samples (0.8 mL) were collected from the retroorbital vessels of the mice, and then used to obtain serum layers (300 μL) by centrifuging them for 20 min at 4 °C at a speed of 4000 rpm. To determine oxidative stress levels, blood serum and hippocampal tissue samples were obtained from the mice. The levels of malondialdehyde (MDA), glutathione peroxidase (GSH-Px), catalase (CAT), nitric oxide (NO), superoxide dismutase (SOD), and corticosterone and total antioxidant capacity (T-AOC) were measured using a kit from the Nanjing Jiancheng Bioengineering Institute.

### Immunofluorescence

Animals were sacrificed after they were treated with CUMS and/or music for 28 days and behavioral tests. These were achieved by intracardial perfusion with saline, and the brains of the mice were fixed with 4% paraformaldehydeFixed brains were dehydrated once in 20% sucrose and twice in 30% sucrose (both in phosphate-buffered saline [PBS]). The coronal sections were cut to a thickness of 35 μm, and washed with 1× PBS. The sections were blocked with a blocking buffer (1% BSA (Bovine Serum Albumin) + 0.3% Triton X-100 + 10% goat serum in PBS) for one hour at room temperature after three washes with 1× PBS. The coronal sections were then incubated with the primary antibody rabbit anti-doublecortin (DCX, Cell Signaling Technology, #14082, 1:400), rabbit anti-glial fibrillary acidic protein (GFAP, Cell Signaling Technology, #14082, 1:400), mouse anti-ionized calcium-binding adapter molecule 1 (IBA-1, Cell Signaling Technology, #14082, 1:400), and rabbit anti-microtubule association protein-2 (MAP2, Cell Signaling Technology, #14082, 1:400) additionally for an overnight at 4 °C, followed by an additional two hours at room temperature with fluor conjugated secondary antibodies of goat anti-rabbit IgG (Alexa Fluor® 594 conjugate, Invitrogen, #A11008, 1:1000) and goat anti-mouse IgG (Alexa Fluor® 594 conjugate, Invitrogen, #A21422, 1:1000). The sections were washed three times and stained with 4′,6-diamidino-2-phenylindole solution. Finally, images were captured using a Leica TCS SP8 confocal microscope (Leica Microsystems, Germany) [39].

### Data analysis

GraphPad Prism software (version 5.0) was used for statistical analysis. One-way ANOVA followed by Tukey’s multiple comparisons test was used to assess differences between the groups. *P* < 0.05 was regarded as statistically significant, and all data are presented as mean ± standard error of mean.

## Results

### Music prevents depression and anxiety-like behavior in CUMS mice

Behavioral tests are the most effective means of assessing visual response to depression- and anxiety-like behavior in mice to verify whether music can prevent depression- and anxiety-like behaviors caused by chronic high-intensity stress. We established a CUMS+Music mouse model by subjecting the mice to high-intensity stimulation during the day and listening to music for relaxation at night. In this way, we simulated the conditions of humans who are under intense stress during the day owing to factors such as work or family and relax by listening to music at night.

**Fig. 1:**
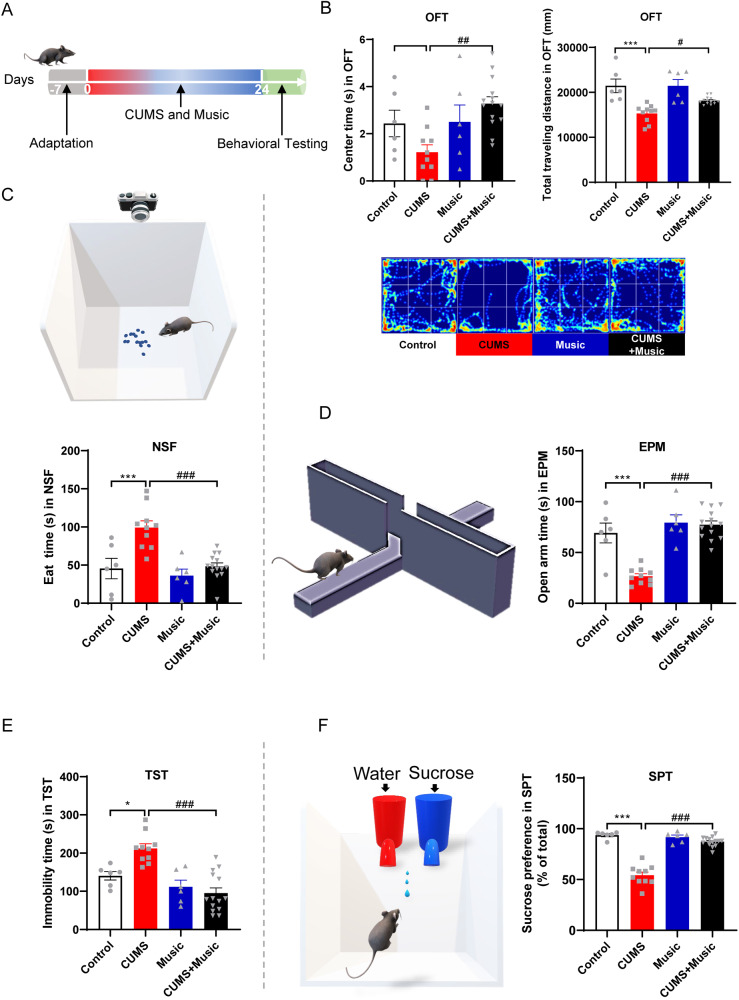
Music prevents depression and anxiety-like behavior in CUMS mice. **A** Experimental design: schematic representation of the music prevention. **B** Music prevents the decreased explore central area time and total traveling distance induced by CUMS in the open field test. **C** Music prevents the increase of preparation time to eat the food induced by CUMS in the novelty-suppressed feeding test. **D** Music inhibited the times decrease of open arms entries induced by CUMS in the elevated plus maze test. **E** Music prevents the increase of immobility times induced by CUMS in the tail suspension test. **F** Music prevents the decreased consumption of sucrose solution induced by CUMS in the sucrose preference test. All values are presented as means ± SEM. Control or Music *n* = 6, CUMS *n* = 10, CUMS + Music *n* = 14. *^(#)^*P* < 0.05; **^(##)^*P* < 0.01; ***^(###)^*P* < 0.001. OFT open field test, NSF novelty-suppressed feeding, EPM elevated plus maze, TST tail suspension test, SPT sucrose preference test.

The experimental procedure is illustrated in Fig. 1A. CUMS mice that were stimulated only showed shorter paths through central areas, a shorter total distance of movement in the OFT (Fig. 1B), whereas CUMS+Music mice that were stimulated and relaxed by music were more active.

Animals with depression show decreased interest in food intake and appetite. Novelty-suppressed feeding (NSF) experiments can be used to observe changes in animals without euphoria. CUMS mice required more time to eat food in the novel environment than CUMS+Music mice (Fig. 1C).

The EPM is used to evaluate the anxiety response of rodents. When mice face a new condition (open arm), they are curious to explore; when they are kept in a dark environment (closed arm), conflict between the two occurs, resulting in anxiety. The height of the entire maze from the ground for animals is equivalent to that of the edge of a cliff for humans, which can lead to fear and anxiety in animals. The anxiety behavior of the mice was evaluated by comparing the retention time and distance covered in the open and closed arms. CUMS+Music mice showed a greater ability to explore, while CUMS mice preferred to hide in a dark and claustrophobic environment (Fig. 1D).

In the tail suspension experiment, the animals were hung with their heads facing downward, and to overcome the abnormal body position, they first struggled to escape. When they were unable to get out of the situation, they became intermittently motionless, showing “behavioral despair.” CUMS+Music mice did not exhibit despair, whereas CUMS mice exhibited severe behavioral despair (Fig. 1E).

Rodents have a strong natural desire for sweet foods and will selectively drink the sweetened solution when given a free choice between two drinks such as a sucrose solution and plain water. All groups in this experiment, except the CUMS group, showed a preference for sucrose solution. However, the CUMS group in this experiment was not inclined to drink the sucrose solution because of the depression-like behavior caused by chronic stress (Fig. 1F). Overall, the CUMS+Music mice did not exhibit depression-like or anxious behavior, even when subjected to high levels of stress.

### Music prevents oxidative stress in serum and brain tissues of mice

Reactive oxygen species (ROS) have been suggested to play a role in depression [40]. We investigated whether music can regulate antioxidant capacity and ROS levels. We also indirectly assessed the levels of antioxidant enzymes [11].

NO and MDA levels are often measured to determine the damage caused by oxidative stress [41]. SOD is a vital component of the antioxidant enzyme system in biological systems. SOD exhibits an extremely strong anti-inflammatory effect. GSH-Px is an important peroxidase enzyme widely present in organisms. CAT is an antioxidant enzyme found in almost all organisms. Its main role is to catalyze the breakdown of hydrogen peroxide into water and oxygen and to remove hydrogen peroxide from the body, thus protecting cells from the toxic effects of hydrogen peroxide. It is one of the key enzymes of the biological defense system and provides an antioxidant defense mechanism for the organism [42]. T-AOC refers to the total antioxidant levels of various antioxidant substances and enzymes and can be used to evaluate the antioxidant capacity of bioactive substances.

**Fig. 2:**
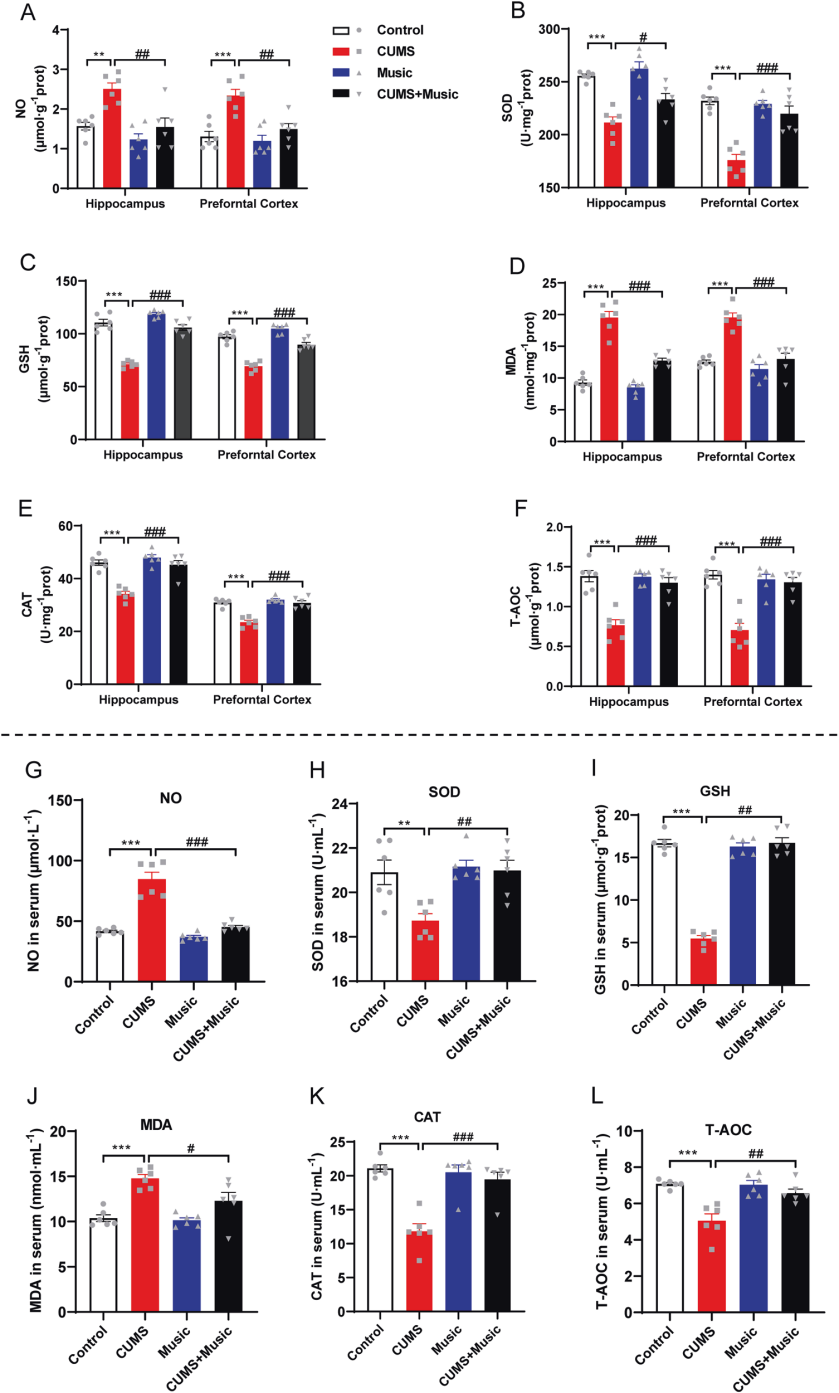
Music prevents oxidative stress in serum and brain tissues of mice. **A**–**L** Music prevents abnormal expression of oxidative stress-related factors such as NO, SOD, GSH, MDA, CAT, T-AOC in the blood or hippocampus and prefrontal cortex of CUMS. All values are presented as means ± SEM. *n* = 6 per group. *^(#)^*P* < 0.05; **^(##)^*P* < 0.01; ***^(###)^*P* < 0.001. NO nitric oxide, SOD superoxide dismutase, GSH glutathione peroxidase, MDA malondialdehyde, CAT catalase, T-AOC total antioxidant capacity.

The results showed that NO, SOD, GSH-Px, MDA, and CAT expression levels and T-AOC were abnormal in the serum (Fig. 2A–F) and cortical and hippocampal tissues (Fig. 2G–L) of CUMS mice compared to those of the control mice, while no abnormalities were found in CUMS+Music mice.

### Music prevents elevated levels of inflammatory factors in serum and brain tissues of mice

**Fig. 3:**
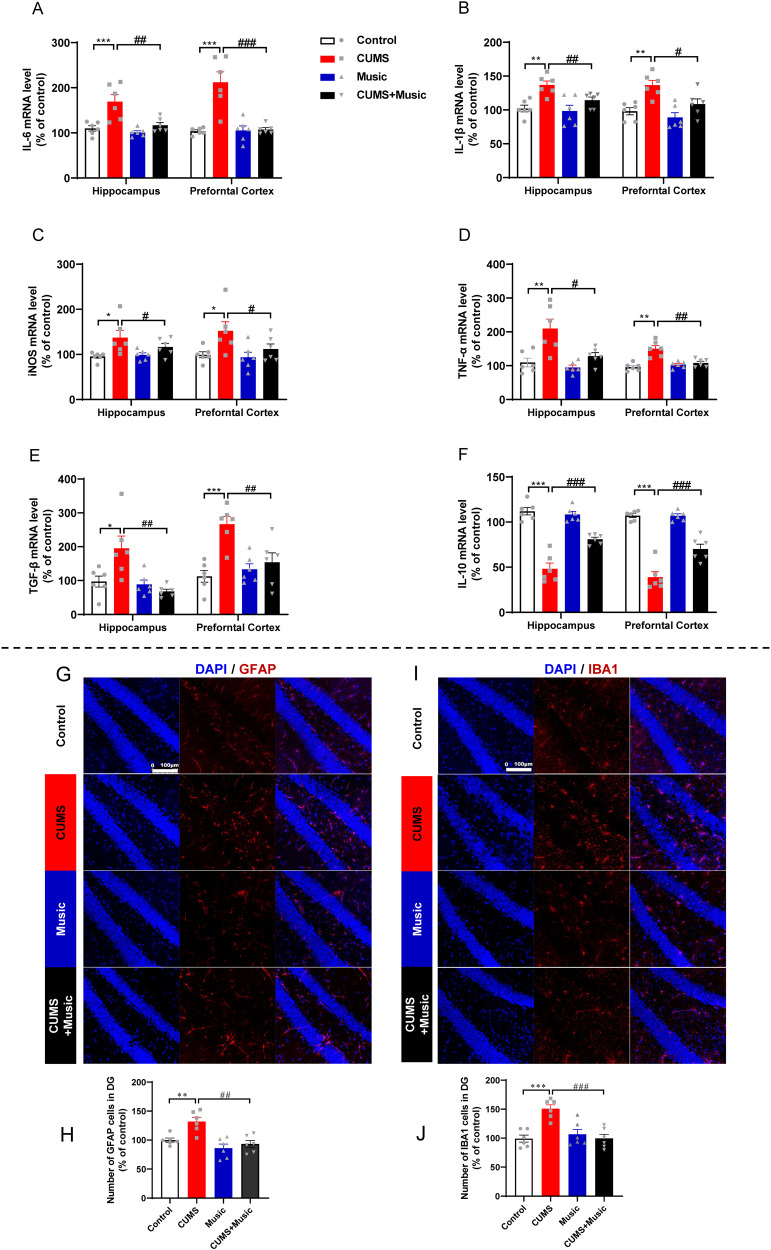
Music prevents elevated levels of inflammatory factors in serum and brain tissues of mice. **A**–**F** Music prevents abnormal expression of inflammation-related factors such as IL-6, IL-1β, iNOS, TNF-α, TGF-β, IL-10 in the hippocampus and prefrontal cortex of CUMS. **G**, **H** Representative fluorescence micrographs showing the morphology and density of microglia in DG. **H** Music prevents the increase of microglia in the DG of CUMS. **I**, **J** Representative fluorescence micrographs showing the morphology and density of astrocyte in DG. **J** Music prevents the increase of astrocytes in the DG of CUMS. All values are presented as means ± SEM. *n* = 6 per group ^*(#)^*P* < 0.05; ^**(##)^*P* < 0.01; ^***(###)^*P* < 0.001. IL-6 interleukin 6, IL-1β interleukin 1 beta, iNOS inducible nitric oxide, TNF-α tumor necrosis factor-α, TGF-β transforming growth factor-β, IL-10 interleukin 10, DAPI 2-(4-Amidinophenyl)-6-indolecarbamidine dihydrochloride, GFAP glial fibrillary acidic protein, IBA1 ionized calcium-binding adapter molecule 1.

**Fig. 4:**
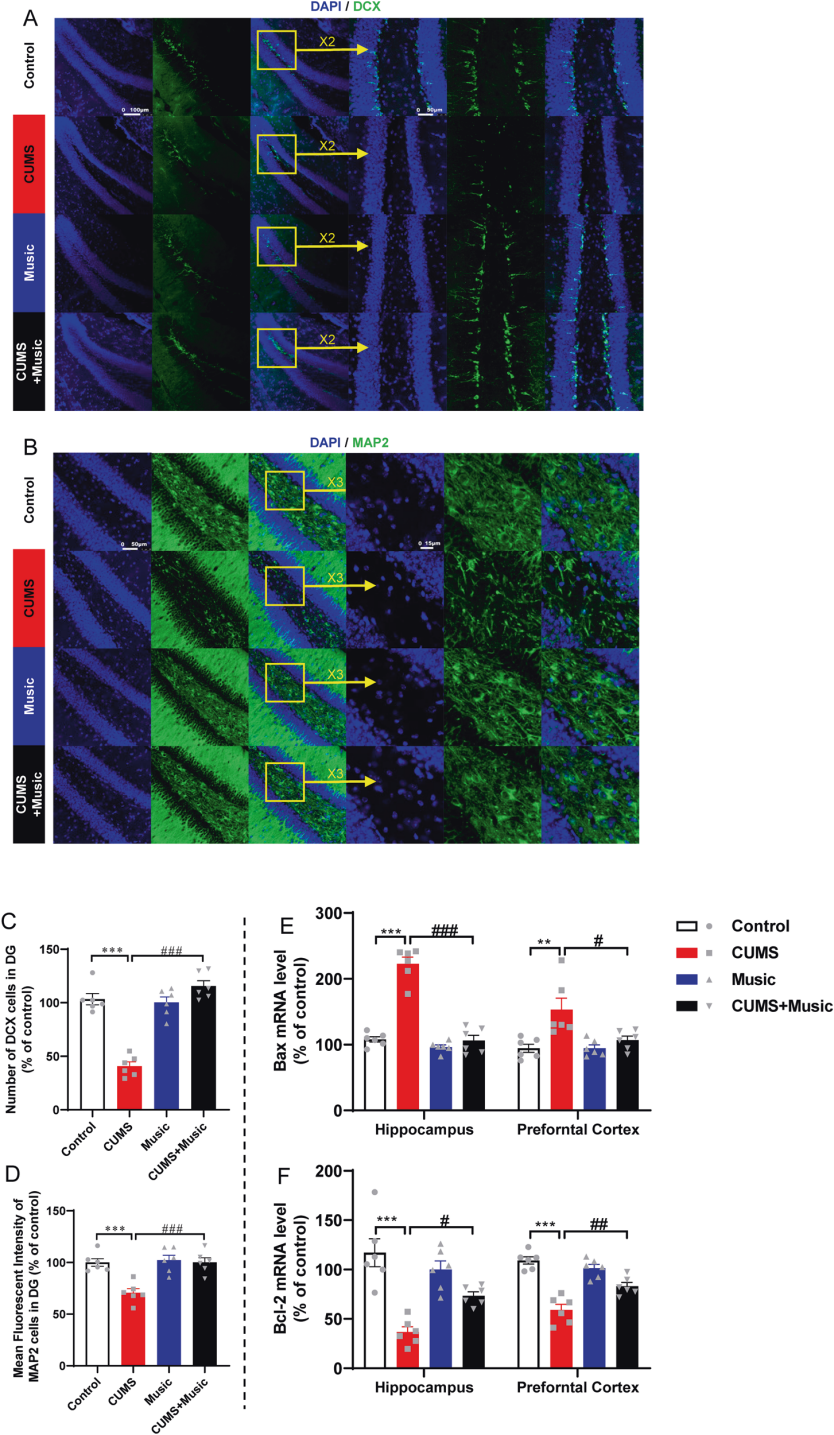
Music prevents neuronal death and promotes neurogenesis. **A**, **B** Representative fluorescence micrographs showing the morphology and density of immature neurons and neurons in DG. **C** Music prevents the loss of DCX cell induced by CUMS in DG. **D** Music prevents the loss of MAP2 cell induced by CUMS in DG. **E**, **F** Music prevents the abnormal expression of apoptosis-related factors such as Bax, Bcl-2 in the hippocampus and prefrontal cortex of CUMS. All values are presented as means ± SEM. *n* = 6 per group. *^(#)^*P* < 0.05; **^(##)^*P* < 0.01; ***^(###)^*P* < 0.001. DAPI 2-(4-Amidinophenyl)-6-indolecarbamidine dihydrochloride, DCX doublecortin, MAP2 microtubule association protein-2, Bax B-cell lymphoma-2 associated X protein, Bcl-2 B-cell lymphoma-2.

Depression is a long-lasting and persistent mood disorder in which the regulatory mechanisms of neuroinflammation play a contributing role to the physiopathology. To verify whether music can prevent abnormal expression of inflammatory factors, we examined the RNA expression levels of interleukin 6 (IL-6), interleukin 1 beta (IL-1β), inducible nitric oxide (iNOS), tumor necrosis factor-α (TNF-α), and transforming growth factor-β (TGF-β) in mouse cortical and hippocampal tissues (Fig. 3A–E). The results showed that inflammatory factor levels were elevated in CUMS mice compared to that in controls, and those in CUMS+Music mice were essentially similar to those in controls. We also examined the expression of the inflammatory suppressor interleukin 10 (IL-10), which was downregulated in the CUMS group. IL-10 was upregulated in the CUMS+Music group and was higher than that in the control group (Fig. 4F).

Microglia play a key role in neuroinflammation by initially suppressing the progression of neuroinflammation and subsequently exacerbating the inflammatory response via inflammatory factors and interacting with glial cells, such as astrocytes, to further exacerbate neurotoxicity. In addition, both astrocytes and microglial cells were mediators for neurogenesis in DG, given that microglia in the DG region transfer a microglia-enriched microRNA, miR-146a-5p, via secreting exosomes to inhibit neurogenesis in depression [43]. Furthermore, astrocytes have been found to mediate cholinergic regulation of adult hippocampal DG neurogenesis [44]. We therefore analyzed microglia and astroglia in the DG using immunofluorescence experiments and showed that microglia in the DG of CUMS mice were increased in number and size in the cytosol (Fig. 4I). The number of astrocytes was increased and the level of inflammation was elevated. CUMS+Music mice were similar to control mice in this aspect (Fig. 4G).

### Music prevents neuronal death and promotes neurogenesis

Patients with depression exhibit massive hippocampal neuronal decay and loss [45]. To verify whether music is related to neuronal death, we labeled MAP2 by immunofluorescence to determine the number of neurons. The results showed that the number of neurons in the DG was significantly reduced in CUMS mice, while no significant reduction was observed in CUMS+Music mice (Fig. 4B). Overexpression of Bax (B-cell lymphoma-2 associated X protein), a water-soluble protein homologous to Bcl-2 (B-cell lymphoma-2) and coded by an apoptosis-promoting gene in the Bcl-2 gene family, antagonizes the protective effect of Bcl-2 and leads to cell death. We found decreased Bcl-2 mRNA expression levels (Fig. 4F) and increased Bax mRNA expression levels (Fig. 4E) in the cortical and hippocampal tissues of CUMS mice, whereas CUMS+Music mice were similar to the control mice in this aspect. This suggests that music may inhibit abnormal death of hippocampal neurons.

DCX is a protein specifically expressed in neural precursor cells and newborn neurons. It can also be used to identify early immature neurons. Immunofluorescence results showed that DCX expression was significantly lower in the DG of CUMS mice than of control mice, and there was no significant difference between CUMS+Music and control mice (Fig. 4A).

### Music prevents the HPA axis activation

**Fig. 5:**
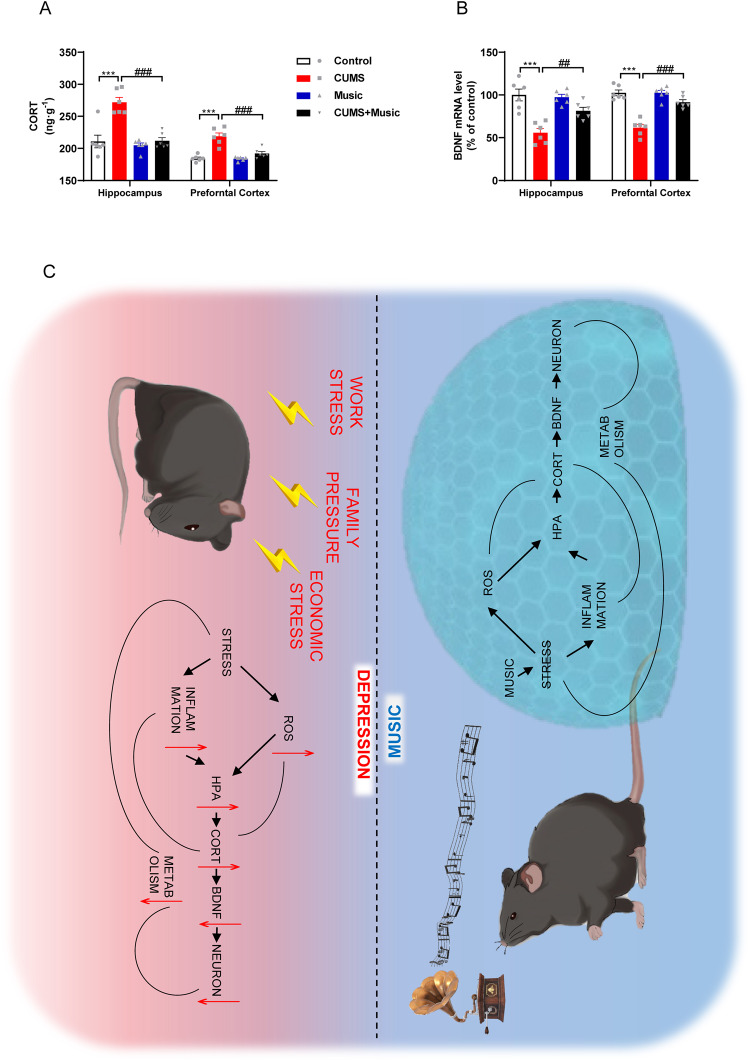
Music prevents the HPA axis activation. **A** Music prevents the increase of corticosterone by CUMS in qRT-PCR. **B** Music prevents CMUS-induced BDNF downregulation. **C** Schematic diagram of how music prevents stress-induced depression and anxiety-like behavior. All values are presented as means ± SEM. *n* = 6 per group. *^(#)^*P* < 0.05; **^(##)^*P* < 0.01; ***^(###)^*P* < 0.001. CORT corticosterone, BDNF brain-derived neurotrophic factor, ROS reactive oxygen species, HPA hypothalamus-pituitary-adrenal.

Brain-derived neurotrophic factor (BDNF) is a member of the nerve growth factor family, and as an important neurotrophic factor, it has neuroprotective functions and plays an important role in the formation of synapses and the maintenance of neural protrusion morphology. Corticosterone decreases BDNF expression in the hippocampus. Thus, decreased BDNF levels may be an important link between corticosterone levels and hippocampal injury. We measured corticosterone expression levels in cortical and hippocampal tissues and showed that corticosterone mRNA expression levels were significantly higher in CUMS mice and not significantly different between control and CUMS+Music mice (Fig. 5A). BDNF mRNA expression was significantly reduced in the cortical and hippocampal tissues of CUMS mice, while CUMS+Music mice were similar to the control mice in this aspect (Fig. 5B).

## Discussion

Previous animal studies have suggested that music alleviates pain and improves social behaviors under various pathophysiological conditions [46–49]. In addition, it has been reported that music adjuvant therapy enhances the efficacy of antiepileptic drugs in various temporal lobe epilepsy models [46]. Interestingly, results from Yang et al. showed that music can induce the opening of blood-brain barrier in mice, suggesting the ability of music to regulate the interaction between the brain and peripheral system [50]. Here we have demonstrated for the first time in mice experiments that music can prevent depression-like behavior. It protects neurons from loss and ensures normal homeostasis by regulating the HPA axis, affecting the release of corticosterone and BDNF levels, and the levels of oxidative stress and inflammation (Fig. 5C). Although mice can use ultrasonic vocalization, the frequency of the music can be received by both mice and humans (less than 20 kHz). In addition, it is known that some sounds and vocalizations are aversive to rodents, but Yan et al. found that none of the different types of music they played, including upbeat, slow, and heavy metal music, caused aversive or fear responses in the mice [51]. These results therefore support the translation of these pre-clinical findings to humans.

Music is often used clinically as an adjunctive treatment for depression and can effectively relieve stress [27, 52]. However, the potential ability of music to prevent depression was not studied before. Here we prevented depression by establishing a CUMS mouse model that was exposed to music for 1.5 h every night to mimic the prevention of depression by listening to music in humans after experiencing high-intensity stress, which was tested behaviorally after 28 days. In the behavioral experiments in which depression-like behavior was assessed, including the OFT, novelty-suppressed feeding, EPM, TST, and SPT, mice that received stimuli and listened to music (CUMS+Music) did not show depression-like behavior. Even in the EPM experiment, mice listening to music (Music and CUMS+Music mice) had longer open arm duration than normal mice, which seems to indicate that music can prevent mice from developing depression-like behaviors and that listening to music can render mice more active.

Depression is the most prevalent psychiatric disorder worldwide; however, its neural mechanism remains elusive. Although we do not fully understand how the brain resists stress, the most influential factor is the “stress hypothesis” [53]. This hypothesis suggests that the hypothalamus, pituitary, and adrenal glands comprise the neuroendocrine regulatory system, also known as the HPA axis. The HPA axis is involved in controlling the response to stress and coordinating many physical activities. Stress induces inflammation and oxidative stress to over-activate the HPA axis, leading to the release of large amounts of corticosterone [54]. Corticosterone is a glucocorticoid secreted by the adrenal cortex. Under stress and aging conditions, elevated plasma corticosterone levels can lead to morphological and functional damage in the hippocampus [55]. Corticosterone can cause a decrease in the expression of BDNF in the hippocampus, an important neurotrophic factor that has neuroprotective functions and plays an important role in the formation of synapses and maintenance of neuroprotrusive morphology [56, 57]. Decreased BDNF levels are believed to be a direct cause of depression [58]. In consistent with the above hypothesis and findings, here we found increased corticosterone expression and substantial depletion of BDNF in the hippocampus and cortex of CUMS mice, whereas no abnormalities were found in CUMS+Music mice. This demonstrated that music protects the HPA axis from damage. Decreased BDNF in the brain is often accompanied by neuronal loss. Therefore, we examined the number of newborn neurons and neurons in the DG of the hippocampus and examined the mRNA levels of the apoptosis-affecting factors Bax and Bcl-2, showing that CUMS+Music mice do not suffer from significant neuronal loss.

Depression is a chronic inflammatory and oxidative stress disorder caused by stressors and the HPA axis plays a key role in the development of depression [59, 60]. To verify whether music protects CUMS mice from depression-like behaviors by altering oxidative stress levels, we examined the expressions of NO, SOD, GSH, MDA, CAT, and GSH-Px in the mouse hippocampus, cortex, and serum. Unsurprisingly, mice exposed to both stress and music (CUMS+Music) did not show abnormalities in the oxidative stress indicators, whereas mice exposed only to stress (CUMS) showed severe oxidative stress disorders. This suggests that music protects mice from the oxidative stress disorders caused by stress overload. Excessive stress not only disrupts oxidative stress but also causes an increase in inflammatory factor levels. For validation of inflammatory factor level, we examined the expression of mRNAs of inflammatory factors such as IL-6, IL-1β, iNOS, TNF-α, and TGF-β in the cortex and hippocampus. We also examined the activation/number of microglia and astrocytes in the DG, which indirectly reflect the level of inflammation in the brain [61]. These results suggest that music may prevent abnormal neuroinflammation. For example, the mRNA expression levels of pro-inflammatory factors such as IL-6, IL-1β, iNOS, TNF-α, and TGF-β were upregulated in the cortex and hippocampus of CUMS mice, and those of the anti-inflammatory factor IL-10 were downregulated. In addition, increased numbers of microglia and astrocytes are accompanied by increased levels of inflammation, and CUMS mice had a higher number of cells in the hippocampal DG region. No abnormalities were observed in CUMS+Music mice. These results suggest that music may be effective in preventing elevated neuroinflammation caused by stressful stimuli.

Although we have provided strong pre-clinical evidence for a beneficial effect of music to prevent depression, there are some limitations in this study. The first limitation of this study is that we used only male mice to mimic human conditions. However, it is very likely that music could also prevent female mice from stress-induced depression, and this hypothesis needs validation with future investigations. Secondly, the mice are nocturnal animals. Ideally, the mice should be subjected to the CUMS at night and/or listening to the music during the day. However, it is well known that most researchers performed rodent experiments during the day. This phenomenon is largely due to the inconvenience to perform experiments at night, especially experiments that may need a certain period of time. The CUMS establishment experiments need a long time to complete each day, and thus it is very difficult for the researchers to perform CUMS experiments at night. Nevertheless, with proper controls, it is very unlikely that this would affect the soundness of the conclusion found in this study. Finally, the gene expression profile in auditory cortex of mice in response to music has not been evaluated, and future studies using RNA-seq analysis are necessary to strengthen our understandings on the beneficial effects of music.

In conclusion, the neural mechanism by which music prevents anxiety- or depression-like behavior may be that music protects against the disruption caused by oxidative stress and increase in inflammation levels caused by severe stress in mice and prevents the HPA axis from releasing large amounts of corticosterone due to overexcitation, which leads to the downregulation of BDNF. Finally, it protects neurons from loss and maintains homeostasis. Therefore, we have demonstrated for the first time in animal studies that music is effective in preventing depression-like behavior, which provides substantial evidence for clinical studies. In future, music should be explored as a more natural method of preventing depression.
